# Circulating Cell-Free DNA-Based Methylation Pattern in Saliva for Early Diagnosis of Head and Neck Cancer

**DOI:** 10.3390/cancers14194882

**Published:** 2022-10-06

**Authors:** Natalia Birknerova, Veronika Mancikova, Evan David Paul, Jan Matyasovsky, Pavol Cekan, Vladimir Palicka, Helena Parova

**Affiliations:** 1 Department of Clinical Biochemistry and Diagnostics, Faculty of Medicine in Hradec Kralove and University Hospital, Charles University, 50005 Hradec Kralove, Czech Republic; 2 MultiplexDX s.r.o., Comenius University Science Park, Ilkovicova 8, 84104 Bratislava, Slovakia; 3 MultiplexDX Inc., One Research Court, Suite 450, Rockville, MD 20850, USA

**Keywords:** biomarker, cell-free DNA (cfDNA), circulating tumor DNA (ctDNA), DNA methylation, early cancer detection, head and neck cancer, liquid biopsy, saliva

## Abstract

**Simple Summary:**

Liquid biopsy represents a promising alternative to standard-of-care tissue biopsies, outperforming the latter in several aspects: invasiveness, cost, spatial limitation to a single region, and time to result. The pursuit of knowledge regarding the detection and analysis of circulating tumor DNA, especially methylation profiling, represents a unique opportunity for real-time detection and monitoring of tumor properties. Despite advances, most head and neck cancer patients are still diagnosed at an advanced stage, resulting in a poor prognosis. This review aims to synthesize our current understanding of DNA methylation changes in squamous cell head and neck cancer as a potential disease biomarker and to identify gaps for further research.

**Abstract:**

Head and neck cancer (HNC) remains one of the leading causes of mortality worldwide due to tumor diagnosis at a late stage, loco-regional aggression, and distant metastases. A standardized diagnostic procedure for HNC is a tissue biopsy that cannot faithfully portray the in-depth tumor dynamics. Therefore, there is an urgent need to develop simple, accurate, and non-invasive methods for cancer detection and follow-up. A saliva-based liquid biopsy allows convenient, non-invasive, and painless collection of high volumes of this biofluid, with the possibility of repetitive sampling, all enabling real-time monitoring of the disease. No approved clinical test for HNC has yet been established. However, epigenetic changes in saliva circulating cell-free DNA (cfDNA) have the potential for a wide range of clinical applications. Therefore, the aim of this review is to present an overview of cfDNA-based methylation patterns in saliva for early detection of HNC, with particular attention to circulating tumor DNA (ctDNA). Due to advancements in isolation and detection technologies, as well as next- and third-generation sequencing, recent data suggest that salivary biomarkers may be successfully applied for early detection of HNC in the future, but large prospective clinical trials are still warranted.

## 1. Introduction

**Figure 1:**
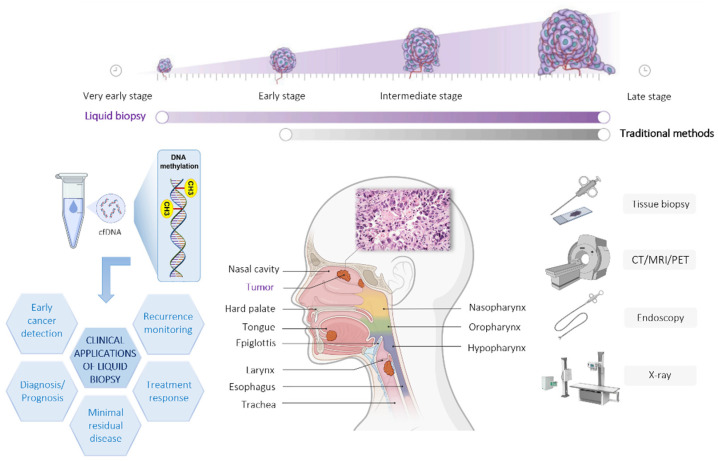
General overview of different cancer screening approaches.

Head and neck cancer (HNC), the sixth most prevalent malignancy worldwide, represents a heterogeneous group of epithelial tumors [1]. In 90% of the cases, head and neck cancer develops from the mucosal epithelium in the oral cavity, pharynx, larynx, and more rarely, the nasal cavity. These cases are referred to as squamous cell carcinomas [2]. The remaining 10% develop from other types of cells, and include lymphomas, adenocarcinomas, and sarcomas [3]. Due to the increasing incidence of cases and numerous alarming factors, such as late diagnosis, loco-regional aggressiveness, and distant metastatic spread, head and neck squamous cell carcinoma (HNSCC) remains one of the leading malignancies with poor survival rate over the last decades [4]. Besides traditional risk factors, alcohol, and tobacco consumption, human papillomavirus infection and Epstein–Barr infection have been identified as additional risk factors for developing cancer in the oropharynx and nasopharynx [5,6]. Because of non-specific “common cold”-like symptoms, such as sore throat, cough, changes in voice, dysphagia, headaches, and white or red patches in the mouth, in most cases, HNSCC is diagnosed in advanced stages [7]. Traditional diagnostic strategies such as physical exams, endoscopies, imaging tests, such as CT (Computed Tomography) scans, MRIs (Magnetic Resonance Imaging), X-rays, PET scans and tissue biopsies, are not sufficient for an early diagnosis [8]. Indeed, while tissue biopsy remains the standard of care, it generally does not provide a comprehensive insight into the tumor dynamics. Therefore, the identification of new diagnostic and prognostic approaches is crucial for early detection and improvement of overall survival (Figure 1).

In recent years, liquid biopsy has drawn an expanding interest in cancer identification and treatment management because of its huge benefits and wide application possibilities. In contrast to traditional tissue biopsy, the liquid biopsy approach focuses on detection of tumor-derived components in bodily fluids [9]. Thus, it is painless, easily repeated, and can give helpful insight into the tumor characteristics and treatment response in a near real-time setting. Furthermore, liquid biopsy can potentially provide a more complete molecular snapshot of the tumor, preventing biopsy results from being affected by intratumor heterogeneity and sampling bias [10] (Figure 1).

Saliva is a multicomponent biofluid reflecting blood content. Most of the components, such as DNA, RNA, proteins, metabolites, hormones, microbiota, etc. are present at comparable levels in both bodily fluids. The components of saliva may be delivered from plasma through various processes like ultrafiltration through gap junctions, passive diffusion, transduction or cell secretion [11]. Saliva plays a crucial role in lubrication, mastication, swallowing, digestion, and it also has antimicrobial activity. It is produced by three main paired glands: parotid, submandibular and sublingual glands [12]. Together with the other labial, buccal, lingual, and palatal minor glands, they can produce as much as 1000–1500 mL of saliva per day. Saliva, as a potential source for liquid biopsy of HNC patients, has a few advantages compared to other body liquids. Saliva mirrors any genomic, epigenomic, proteomic, metabolomic, and transcriptomic changes in circulating analytes, and also provides real-time monitoring of HNC patients [13]. Moreover, saliva is readily available and can be self-collected non-invasively and in high volumes, painlessly, and repeatedly in short intervals, and it contains the clinically relevant information. The potential utility of saliva as a liquid biopsy tool for HNC diagnosis, prognosis, and therapy monitoring is being progressively studied. Currently, the most widely recognized components for liquid biopsy of HNCs include cell-free tumor nucleic acids (DNA, mRNA, and miRNAs), extracellular vesicles, circulating tumor cells (CTCs) and salivary metabolites [14]. Despite the clear potential of saliva-derived biomarkers as a diagnostic tool, their utility in clinical practice is limited due to a number of drawbacks [15]. These include the following: (I) low concentration of components, (II) lack of specificity and sensitivity, which may lead to false-positive and false-negative results, (III) challenging distinction between cancer-specific and healthy components, and (IV) deficient optimization and standardization of pre-analytical and analytical procedures [16]. In this regard, extremely accurate and robust detection technology is required.

Here, we provide a comprehensive assessment of the current developments in saliva cell-free DNA (cfDNA) testing and its application in early cancer detection of HNC. Firstly, we describe an overview of the biology of cfDNA, we then summarize the current data on cfDNA methylation biomarkers, and current cfDNA sequencing approaches. We also discuss clinical phases of biomarker development, the limitations, and future perspectives for early HNC detection.

## 2. cfDNA and ctDNA Biology

In 1948, cfDNA was first discovered by Mandel and Metais in plasma and was also subsequently found in other bodily fluids [17]. The release of cfDNA into the circulation may be mediated by different cell death mechanisms such as apoptosis, necrosis and/or active cellular secretion [18]. The size of cfDNA usually reflects the pattern of apoptotic fragmentation; cfDNA produced by apoptosis is typically composed of fragments of approximately ~167 bp, which correspond to ~147 bp of DNA wrapped around a nucleosome plus ~20–90 bp linker fragment [19,20]. However, apoptotic nuclease can also cleave longer fragments representing di-, tri-, or poly-nucleosomes. Besides the mechanism of release, cfDNA fragment size is heavily influenced by different biological and environmental factors, such as age, gender, metabolic state, tissue of origin, smoking, glucose levels, medication status, infections, menstruation, and pregnancy [20,21]. In healthy individuals, cfDNA is primarily released from hematopoietic cells. In cancer patients, a varying proportion of cfDNA is composed of the circulating tumor DNA (ctDNA) released from tumor cells [22]. Detection and analysis of ctDNA is challenging due to its low concentration, high fragmentation, and low yields. Therefore, optimization of appropriate quantitative and qualitative pre-analytical and analytical procedures is important. Differences in ctDNA yield can have a significant impact on the sensitivity of a given assay and must be considered during study design. Thus, the assay’s sensitivity is determined by the amount of ctDNA inputs, the sequencing efficiency, and the appropriate analysis [23]. Notably, several studies have already reported methods for collection, storage, and isolation of cfDNA from different body fluids such as plasma, saliva, urine, cerebrospinal fluid, pleural fluid, and others [16,24].

The actual ctDNA fraction can vary from 0.01% to 60% of whole cfDNA. Eventual concentration levels of ctDNA depend on the tumor volume, location, stage (ranging from ≤0.01–0.1% in early-stage to ≥5–10% in late stage), proliferation rate and vascularization, but are still highly variable among different patients [25]. Additionally, ctDNA levels can also be influenced by surgery, radiotherapy, and chemotherapy treatment [26]. It has been reported that the half-life of ctDNA ranges from 16 min to 2.5 h in circulation, clearly showing that ctDNA can serve as a snapshot of tumor burden in real-time [23].

Given the complexities of cfDNA and ctDNA biology, many factors need to be considered for liquid biopsy experiments, including biological and environmental. Additionally, technological and instrumental limitations, including pre-analytical factors, play a significant role in overall detection and analysis of all circulating DNA.

### Pre-Analytical and Analytical Phase Examination

The pre-analytical workflow begins with a decision concerning the optimal sample type. Saliva represents a unique bodily fluid that can be utilized for assessing biomarkers in early diagnosis of HNC [13]. Saliva is readily available and is easily collected in a non-invasive manner. Alternatively, oral rinse techniques, employing saline solutions to obtain the sample representing the current dysplastic changes in the oral cavity, can be performed [27,28,29]. Both saliva and oral rinse generally accomplish the same outcome of providing the patient sample suitable for further processing, without any need for trained health personnel. Although saliva sample collection seems to be the more prevalent method for obtainment of patient samples [14,30], it is not always applicable. In some cases, the patient diagnosis prevents the possibility of adequate saliva collection and oral rinse realization becomes the sample of choice [31].

As of now, no general assay describing the sample collection process has been established. It has been demonstrated that the majority of errors occurring during laboratory testing are the result of pre-analytical fluctuations [32]. While the volumetric amount of the sample does represent an important metric during sample collection (Table 1), the specific variations in ctDNA assays, ctDNA recovery, type of commercial kit used and overall quality of cfDNA and ctDNA primarily influence the optimization process of sample collection and processing [33].

Apart from sample collection, storage of body fluids plays a significant role in liquid biopsy performance, and expedient transport of patient samples under appropriate storage conditions (e.g., dry ice) is necessary to prevent material degradation. Rapid degradation of saliva components occurs in approximately 15–60 min after sample collection, representing a key drawback of this methodology. To minimize the degradation of nucleic acid components of the sample, the addition of a stabilizing medium or buffer is necessary. Long-term preservation of salivary samples at −80 °C is only possible after sample processing. Here, the sample is centrifuged, the supernatant is separated from the cell pellet, and stabilizing agents are then added to the remnant, which is used in downstream experiments [50,52,60]. Alternatively, cell-free saliva is possible to obtain utilizing a commercial collection kit (e.g., SuperSAL saliva collector, Oasis Diagnostics, Vancouver, BC, Canada; Oragene^®^ DNA Self-Collection Kit, DNA Genotek, Ottawa, ON, Canada) [29,53]. Recently, a new method for rapid collection of salivary nucleic acids was described that omits the centrifugation step and provides comparable results to standard approaches [61].

## 3. cfDNA Methylation Biomarkers for the Early Detection of HNCs

In general, strategies for early diagnosis of cancers in liquid biopsies are mostly based on the detection of cancer-related modifications of ctDNA. Due to the low concentration of ctDNA in bodily fluids, especially in the early stages of the disease, early detection of cancer represents a significant challenge [62]. Currently, the main ctDNA biomarkers include detection of mutations, aberrant methylation, and atypical fragment patterns. Hereafter, we will focus on the ctDNA methylation-based biomarkers and current sequencing approaches to assess the ctDNA methylation as promising tools for early HNC detection.

Over the last decade, epigenetic alterations, mainly aberrant DNA methylation, have been shown to play a significant role in HNSCC [63]. DNA methylation occurs by adding a methyl group to the 5-carbon position of cytosine (5-methylcytosine, 5mC) predominantly in CpG dinucleotides and it is one of the most studied epigenetic alterations. This covalent modification is catalyzed by DNA methyltransferases and the donor of the methyl group is S-adenosyl-L-methionine (SAM). Additionally, in recent years, 5-hydroxymethyl has been identified as an epigenetic DNA modification of cytosine bases. In the process of DNA hydroxymethylation, conversion of 5mC into 5-hydroxymethylcytosine (5hmC), and further to 5-formylcytosine (5fC), and 5-carboxylcytosine (5caC), is catalyzed by TET (ten-eleven translocation) family of dioxygenases. This important DNA demethylation intermediate, dynamically regulates the DNA methylation level and consequently modifies gene expression. Although the purpose of DNA hydroxymethylation is not fully understood, various types of cancer have been associated with a decrease of global hydroxymethylation, suggesting that 5hmC may also serve as an epigenetic biomarker [64,65,66,67]. Nevertheless, DNA methylation as an epigenetic modification will be the main focus of this review.

Methylation of CpG sites regulates gene expression, organ development, aging, tissue differentiation, and carcinogenesis [68]. Cancer-specific DNA methylation changes occur early in tumorigenesis, plausibly even before gene mutations appear [69]. Abnormal DNA methylation such as global hypomethylation, regional hypermethylation at several genomic locations (primarily CpG islands), and direct mutagenesis at methylated cytosines are all factors that contribute to carcinogenesis and tumor progression [70]. It has been demonstrated that DNA methylation changes can be detected in plasma up to four years prior to a conventional diagnosis [71]. In general, such early detection can lead to better treatment outcomes, prevent complications, and improve patient prognosis. Of note, recent studies have evaluated the utility of saliva-based liquid biopsy approaches for early cancer detection, diagnosis, recurrence monitoring, treatment response, and prognosis in HNC patients [14,72]. Indeed, several saliva biomarkers from HNSCC patients have already been described (Table 1), highlighting their potential for non-invasive screening and early detection. The studies included in Table 1 are listed chronologically and were chosen based on particular key search phrases used in Entrez PubMed: head and neck cancer, liquid biopsy, cfDNA/ctDNA, saliva, DNA methylation, and early cancer detection/diagnosis. No additional inclusion/exclusion criteria were used to filter the publications.

### 3.1. Targeted Gene Analyses

Generally, tumor-suppressor genes become partially silenced due to hypermethylation of the promoter region, which has been described for a plethora of genes [50]. Cancerous cells present increased levels of DNA methylation accompanied by signal stability during cell division. This leads to the conclusion that DNA methylation patterns could be studied as potential biomarkers for early cancer detection [39,40]. DNA methylation pattern of HNSCC has been studied previously on a whole-genome range [38,42]. Referenced studies mainly describe aberrant methylation of tumor-suppressor genes, associated with gene silencing leading to the start of the tumorigenesis process. The decision to select particular genes in the individual studies was mainly driven by (a) methylation databases and repositories; (b) methylation arrays; (c) and references back to previous studies. The majority of genes included in the following studies are tumor suppressor genes (TSGs), while simultaneously being hypermethylated in samples of HNC patients compared to control.

The first reports used hypothesis-driven targeted gene analyses to identify the first initial ctDNA methylation markers. While evaluating a limited number of genes in small cohorts of patients, they served as proof-of-principle and set the basis for validation of saliva-based diagnostic and prognostic tests in HNC. Rosas et al. [34] was the first break-through study, which detected aberrant promoter hypermethylation of cancer-related genes in the saliva of HNC patients. Using methylation-specific PCR (MSP), they tested hypermethylation of *p16*, *MGMT* and *DAPK* in 30 patients with primary tumors and 30 controls and correlated the results with ctDNA methylation in saliva. Data showed high concordance between methylation patterns detected in primary tumor and saliva, validating for the first time the clinical utility of this biofluid usage in HNC. Subsequently, a study by Righini at al. [35] assessed the methylation of a slightly larger panel of genes (*TIMP3*, *ECAD*, *p16*, *MGMT*, *DAPK*, *RASSF1*, *p15*, *p14*, *APC*, *FHIT*, and *hMLH1*) in primary tumors, normal mucosa samples and saliva from 90 patients. The methylation status of six genes (*TIMP3*, *ECAD*, *p16*, *MGMT*, *DAPK*, and *RASSF1*) showed concordant results in tumors and paired saliva samples using MSP. While evaluating a limited number of genes in small cohorts of patients, these first reports served as proof-of-principle and set the basis for validation of saliva-based diagnostic and prognostic tests in HNC.

In further studies, DNA methylation of two individual genes (*EDNRB* and *RASSF1A*) was frequently validated by various authors, highlighting their potential as HNC biomarkers. The first of these genes, Endothelin receptor type B (*EDNRB*) gene encodes a G-protein-coupled receptor. The interaction of this receptor with endothelins leads to the activation of a phosphatidylinositol-calcium second messenger system [49]. In carcinogenesis, *EDNRB* hypermethylation results in alteration of the ET-1 pathway, which leads to proliferation, angiogenesis and metastasis [73,74,75]

The *EDNRB* gene was shown to be silenced by promoter hypermethylation in saliva samples of HNSCC patients using qMSP in a handful of studies [40,49,63]. Demokan et al. [41] reported the potential to assess *EDNRB* and *KIF1A* hypermethylation for screening of HNSCC with 77.4% sensitivity and 93.1% specificity. Another study [40] demonstrated association of *EDNRB* hypermethylation with histologic diagnosis of premalignancy and malignancy. Schussel et al. [49] analyzed salivary rinses of 191 HNSCC patients and identified hypermethylation of *EDNRB* as well as 8 additional genes. The authors found significant *EDNRB* and *DCC* hypermethylation to be associated with the HNSCC diagnosis with 75% sensitivity and 48% specificity, confirming previous results [40]. All the mentioned studies clearly show the potential of *EDNRB* hypermethylation in salivary rinses to serve as a biomarker to identify patients with premalignant and malignant lesions of HNSCC.

*RASSF1A* is a tumor suppressor gene, whose hypermethylation in the promoter region contributes to its epigenetic inactivation. The mechanism has been described in various cancers, including HNSCC [76]. A few studies confirmed *RASSF1A* hypermethylation in saliva samples of patients with HNSCC compared to controls. In 2012, Ovchinnikov et al. [46] analyzed methylation events in the promoter of *RASSF1A*, *DAPK1*, and *p16* genes in 143 patients with HNSCC and 46 controls. The panel of these genes could discriminate patients in early stages of HNSCCs from controls (80% sensitivity and 87% specificity). Another study [52] described salivary DNA methylation in five tumor suppressor genes (*RASSF1α*, *p16^INK4a^*, *TIMP3*, and *PCQAP/MED15*) to discriminate and diagnose HPV-positive and HPV-negative HNSCC patients from healthy controls. Interestingly, their results showed higher salivary DNA methylation levels for *RASSF1α*, *p16^INK4a^*, *TIMP3* and *PCQAP/MED15* genes in HPV-negative HNSCC patients (n = 88) compared to the control group (n = 122). Conversely, presence of HPV infection leads to a decrease of the methylation level in cancer patients. Similarly, Gonzalez-Perez study [59] demonstrated that salivary promoter hypermethylation of *RASSF1A* and *p16^INK4A^* genes could be useful for diagnosis of patients with oral squamous cell carcinoma (OSCC).

Furthermore, a collection of studies has focused on identifying and validating DNA methylation gene panels in saliva samples as tests for HNC. Carvalho et al. [39] collected 211 salivary rinses from HNSCC patients and 527 samples from healthy controls and the methylation status of 21 genes was analyzed using methylation specific Q-PCR. They reported a 5-gene diagnostic panel (*CCNA1*, *DAPK*, *DCC*, *MINT31*, and *p16*), which identified HNSCC patients with 34.1% sensitivity and 91.8% specificity.

Next, two studies focused on a quadruple methylation marker diagnostic panel. Nagata et al. [43] were the first to describe MultiNA Microchip electrophoresis System for the semiquantitative analysis of MSP data from 34 patients with OSCC and 24 healthy controls. OSCC was detected with 100% sensitivity and 87.5% specificity using a combination of methylation data of *ECAD*, *TMEFF2*, *RARβ*, and *MGMT* genes and with 97.1% sensitivity and 91.7% specificity using a combination of *ECAD*, *TMEFF2*, and *MGMT*. A newer study reported [56] a methylation marker panel composed of *p16^INK4a^*, *RASSF1A*, *TIMP3*, and *PCQAP/MED15* TSGs, which showed remarkable diagnostic accuracy in the early detection of oral cancer (OC; 91.7% sensitivity and 92.3% specificity) and of oropharyngeal cancer (OPC; 99.8% sensitivity and 92.1% specificity). Their results also showed association of promoter hypermethylation with demographic factors, risk factors, and clinicopathological characteristics. Significant promoter hypermethylation of *p16^INK4a^* and *RASSF1A* was observed in advanced OC stages, compared to early OC stages, and additionally in high-grade (grades 3 and 4) OC tumors, compared to low-grade (grades 1 and 2) OC tumors. For OPC, *p16INK4a*, *RASSF1A*, and *TIMP3* TSGs were significantly hypermethylated in high-grade OPC tumors compared to low-grade OPC tumors.

Apart from DNA hypermethylation mostly affecting promotor regions, DNA hypomethylation, mainly occurring in repetitive elements, is also a hallmark of cancer. Puttipanyalears et al. [48] used a combined bisulfite restriction analysis (COBRA) to identify association between *ALU*-methylation levels and cancer development of OSCC in different groups of tobacco users. Levels of methylation decline from normal to light smoker, heavy smoker and to oral cancer in oral rinse samples. *ALU* hypomethylation thus might also be a beneficial marker for oral cancer screening in oral rinses.

### 3.2. Genome-Wide Methylation Analyses

As opposed to carefully designed targeted gene assays, genome-wide approaches allow to explore DNA methylation in a more comprehensive way and uncover unexpected associations. A pioneer to perform such an investigation was Viet et al. [38] who analyzed a panel of 1505 CpG loci in 807 cancer-related genes using tissue and saliva samples of OSCC patients and found a spectrum of genes involved in cell signaling (*GABRB3*, *IL11*, *NOTCH3*, *NTRK3*, and *PXN*), cell differentiation (*IL11*, *NOTCH3*, and *NTRK3*), development (*INSR*, *NTRK3*, and *PXN*), regulation of transcription (*NOTCH3*) and cell adhesion (*PXN*) to be differentially methylated in preoperative and postoperative saliva samples of OSCC patients compared to normal controls.

Utilizing the Infinium HumanMethylation27 BeadChips (Illumina, San Diego, CA, USA) it was possible to cover an even larger portion of the genome’s CpGs. Several studies [42,51,55,57] analyzed the role of aberrant DNA methylation in oral rinses of head and neck squamous cell carcinoma and healthy controls with this technology. The first uncovered that hypermethylation of *HOXA9* and *NID2* genes is highly sensitive (94%) and specific (97%) for early detection of OCSCC [42]. The latter study [51] reported three different candidates, *ZNF14*, *ZNF160* and *ZNF420*, for early detection of HNSCC with 100% specificity.

In 2020, Srisuttee et al. [57] uncovered *NID2* promoter methylation as a marker for screening of OSCC. They performed a bioinformatics analysis of methylation microarray data of the Infinium HumanMethylation27 BeadChip, selected the cg22881914 of *NID2* gene and subsequently successfully validated it using qMSP. Another study [55] demonstrated the potential of methylated cg01009664 of the thyrotropin-releasing hormone (*TRH*) gene as a potential biomarker for OSCC and oropharyngeal SCC using oral rinse.

## 4. ctDNA Methylation-Based Sequencing Techniques

### 4.1. Next-Generation Sequencing

Whole-genome bisulfite sequencing (WGBS) is currently the gold standard DNA methylation profiling technology [77]. However, high cost, low recovery of input DNA, and high demands on the sequencing depth make it unsuitable for clinical use. However, there are other next-generation sequencing (NGS) approaches to detect the methylation status that are more attractive for clinical practice. These methods can be divided into (a) bisulfite conversion methods, such as reduced-representation bisulfite sequencing (RRBS), single-cell reduced-representation bisulfite sequencing (scRRBS) for cfDNA [2], and methylated CpG tandem amplification and sequencing (MCTA-seq) [78]; (b) enrichment-based methods, which include methyl-CpG binding domain sequencing (MBD-Seq) [79], methylated DNA immunoprecipitation sequencing (MeDIP-Seq) [80] and improved technology for cfDNA called methylated DNA immunoprecipitation sequencing (cfMeDIP-Seq) [81]; and (c) restriction enzyme-based methods, which take advantage of methylation-sensitive restriction enzymes combined with the subsequent sequencing of size-selected DNA fragments (MRE-Seq) [82].

Recent advances in technology, such as methyl-BEAMing, enhanced linear-splinter amplification sequencing (ELSA-Seq), cfMeDIP-seq, and scRRBS can help improve the application of cfDNA methylation sequencing by reducing the requirements regarding the amount of DNA input and increasing analytical sensitivity. Unfortunately, NGS approaches still require highly optimized lab workflow and pooling of multiple samples due to cost-effectiveness, which lengthens the turnaround times [83]. Moreover, despite improvement of the advanced bisulfite-based methods, there are several advantages to being able to study DNA methylation from native DNA. Firstly, bisulfite conversion leads to massive degradation and/or loss of DNA and further downstream analysis may thus be affected. Additionally, bisulfite-based sequencing cannot discriminate between 5mC and other modifications such as 5hmC, 5fC, and 5CaC [84,85].

### 4.2. Third-Generation Sequencing: PacBio SMRT Sequencing

Single-molecule real-time (SMRT) sequencing, developed by Pacific Biosciences (PacBio), is the first nanosensor-based technology with the capability to directly detect DNA modifications, including N6-methyladenine, 5-methylcytosine, and 5-hydroxymethylcytosine. Detection of modified bases occurs without any prior chemical/enzymatic conversions and PCR amplification by using alterations in the kinetic signals of a DNA polymerase [86,87,88].

**Figure 2:**
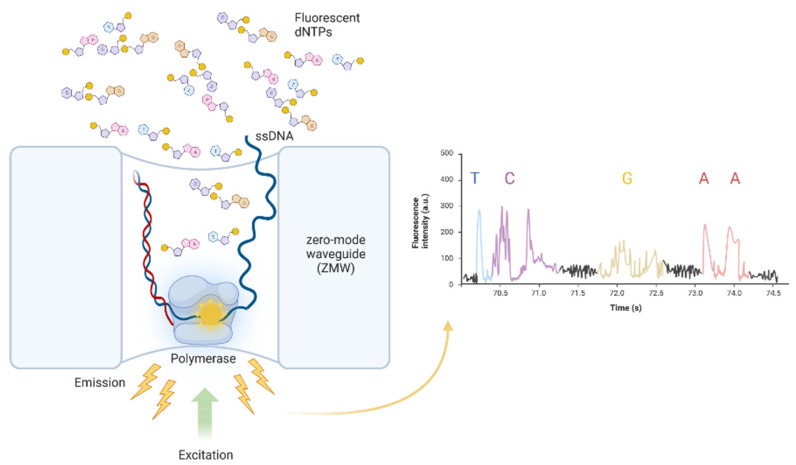
Scientific background of PacBio SMRT sequencing.

In SMRT sequencing, the DNA polymerase synthesizes a new complementary DNA strand by incorporating different uniquely fluorescently-labelled nucleotides, and the fluorescent signal is recorded in real-time in the zero-mode waveguides (ZMWs) [89] (Figure 2). The library is created by ligating hairpin adapters to double-stranded DNA creating circular DNA templates. Hairpin adapters are used to anneal sequencing primers to circularized DNA templates. Prior to sequencing, DNA polymerase bound to a DNA template is immobilized to the bottom of the ZMWs. The error rate for a single pass is ~13% and mainly consists of single nucleotide indels. However, since the utilized DNA template is circular, the error rate is reduced with each pass of the DNA polymerase, which is especially useful for long-read sequencing [90].

Pulse signals in SMRT sequencing, which are associated with nucleotide polymerization, include the interpulse duration (IPD) and the pulse width (PW). IPD represents the time interval between two consecutive base incorporations and PW is characterized by the emission signal related to base incorporation [88,91]. Because the changes in the kinetic signal caused by 5mC modification are extremely subtle, robust genome-wide measurement of 5mC modification is very challenging [91]. The first study concerning detection of 5mC using SMRT sequencing took advantage of the enhancement of the kinetic signature of 5-carboxylcytosine upon conversion of 5mCs using Tet1 [92]. Following this, Tse et al. [91] attempted to develop a holistic kinetic (HK) model to improve the accurate detection of 5mC using SMRT sequencing. Based on the validation datasets generated using amplified DNA and DNA treated with M.SssI (the CpG methyltransferase which methylates the C5 position of all CpG sites in a double-stranded DNA), the HK model dramatically improved 5mC detection rates by 90% at 94% specificity. This model was used for different types of extracted DNA, such as buffy coats, placental tissues, and tumor tissues. The overall methylation level analyzed by the HK model highly correlated with bisulfite sequencing results (99%); therefore, the HK model constitutes a new, viable approach for studying epigenetic modifications in molecular diagnostic applications.

To date, one study published in May 2022 [93] concerning circulating tumor DNA methylation analysis using SMRT sequencing in hepatocellular carcinoma (HCC), utilizing the HK model described above, was used for the determination of methylation patterns. In this study, plasma DNA molecules from patients with hepatocellular carcinoma (HCC) were sequenced with SMRT sequencing (PacBio), followed by fragment size and methylation analysis. A new metric, called the HCC methylation score, which reflects the number of cfDNA molecules having a methylation pattern associated with cancer, was introduced. Since longer DNA molecules are expected to possess more CpG sites, such a metric provides more information regarding the methylation pattern associated with tissue-of-origin analysis of particular lone plasma DNA molecules. Although the full utility of SMRT sequencing has yet to be fully explored, it has the potential to unlock new possibilities for long cfDNA-based cancer diagnostics.

### 4.3. Third-Generation Sequencing: Oxford Nanopore Technology

**Figure 3:**
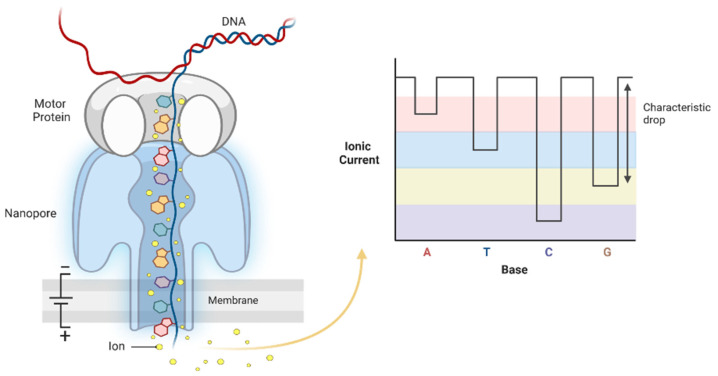
General depiction of ONT sequencing.

Oxford Nanopore Technology (ONT), part of the third-generation sequencing technologies that yield native long reads of single nucleic acid molecules, is a powerful tool for genome-wide profiling of DNA methylation biomarkers. Nanopore sequencing directly detects nucleotides as they pass through a protein nanopore stabilized in an electrically resistant polymer membrane [94]. Sensors detect the ionic current changes shifted by nucleotides occupying the pore in real-time by applying a voltage across this membrane. Of note, the current change can faithfully reflect even nucleotide modification of the sequenced DNA [95] (Figure 3).

Despite ONT sequencing being primarily used for long-read sequencing, several studies showed promising results for plasma ctDNA. ONT sequencing was utilized in the Katsman study [85], which aimed at ctDNA detection and comparison of methylation levels and fragmentation features using ONT and Illumina. Their results showed a high agreement between ONT and Illumina-based WBS and WGBS methods. Nanopore thus represents a reliable alternative to Illumina sequencing, with the advantages of minute instrumentation costs and rapid analysis time [96].

In contrast to other common genome-wide approaches, bisulfite conversion and PCR amplification steps are not required for ONT DNA methylation profiling, which eliminates biases associated with incomplete conversion, DNA fragmentation patterns, or amplification errors. Fast sample prep, sequencing time, and portability of ONT sequencer (MinION) allow for a complete methylation analysis from sample preparation to DNA methylation-based classification in as little as 1–3 h [97,98]. Therefore, the ONT approach could be especially useful for rapid, real-time, and point-of-care clinical liquid biopsy testing.

Despite the advantages of this technology for both research and clinical applications, several drawbacks, such as lower read accuracy, high error rate, frequent kits and reagent modifications, high levels of inter-run variability, and high concentration and quality DNA, still remain [99,100]. However, these limitations are currently being addressed, as the latest data indicate significant improvement in accuracy, read length, and throughput of ONT sequencing [101].

Moreover, Oxford Nanopore technology released a new chemistry modification for short fragment mode (SFM) in March 2022 [102]. This latest release enables users to generate highly accurate information-rich data of any DNA molecule longer than 20 bases using nanopore technology. Additionally, in May 2022 [103], Oxford Nanopore introduced a high-performance and high-accuracy tool for methylation analysis using precise whole-genome PCR-free sequencing. Analysis of epigenetic modifications now runs in parallel with standard base-calling during the experiment. Remora, the new base-calling tool, demonstrated high detection accuracy and quality filtered calls achieving 99.8% accuracy for 5mC in CpG contexts using the most recent Kit 12 chemistry (i.e., Q20+) and R10.4 flow cell. To increase the data yield, ONT also modified the nanopores, motor and run conditions to increase the speed of DNA passing through the nanopore. The latest update contains a new Kit 14 chemistry, which can sequence 420 bases per second, with 99.3% raw read accuracy [104]. Higher data outputs are thus possible with this faster translocation speed, which supports cost effectiveness and experiments that may require higher data volumes.

## 5. Current Challenges and Future Perspectives

In the field of oncology, the analysis of ctDNA methylation has quickly become a promising tool with a wide range of potential clinical uses. However, there are still many hurdles and challenges that must be overcome before its full implementation into clinical practice becomes a reality. The development of reliable, robust, reproducible, sensitive, and specific assays is needed. Additionally, standardization of preanalytical and analytical steps, and uniform operating procedures are pivotal for integration into the clinics in order to minimize false positive/negative results. The inability to isolate and analyse enough DNA molecules from an individual’s biofluids is still a significant barrier to increasing sensitivity. Additionally, the proportion of plasma tumor-derived DNA in cancer patients is typically low, especially in cases of early-stage disease [25].

Biological factors are also crucial to the analytical precision of ctDNA methylation detection, in addition to the limited amount of ctDNA accessible for analysis. It has been established that methylation of CpG sites reflects biological processes that gradually increase in frequency with age and are present in both cancer and normal cells [105]. Other physiological factors that may occur in specific clinical settings could also affect the epigenetic and biological characteristics of cfDNA. Additionally, tumors objectively differ from one another and are even heterogenous within [106]. All the above-mentioned need to be considered when setting up epigenetic-based liquid biopsy assays. Nevertheless, there are already a few examples of non-invasive DNA methylation assays, including liquid biopsy assays, that have been established into commercially available in vitro diagnostic (IVD) tests. The first FDA-approved DNA methylation assay for general colorectal carcinoma (CRC) screening for average-risk adults older than 50 years was *Cologuard^®^ stool-DNA-based test* based on the analysis of the methylation levels of the genes N-Myc downstream-regulated gene 4 (*NDRG4*) and bone morphogenetic protein 3 (*BMP3*) [107,108]. Minimally invasive *Epi proColon^®^ 2.0 test* based on the detection of methylation of Septin9 (*SEPT9*) in plasma was designed to improve adherence of participants to CRC screening [109]. *EarlyTect^®^ CRC test* is an IVD assay that uses cfDNA isolated from 0.5 mL of serum to analyze the methylation status of Syndecan-2 (*SDC2*) [110,111]. Next, liquid biopsy tests are available for breast cancer, capable of prognostication of a specific cancer subtype [112]. *The Therascreen PITX2 RGQ PCR kit* is a methylation-based CE-IVD marked assay that predicts the response to chemotherapy of lymph node-positive, ER-positive, and HER2-negative high-risk breast cancer patients [113]. In lung cancer, increased short stature homeobox gene two (*SHOX2*) methylation level has been identified as a biomarker capable of reliably differentiating between lung tumor tissue and normal tissues associated with early detection in blood plasma, pleural effusions, and bronchial aspirates [114,115,116]. *The Epi proLung BL Reflex Assay^®^* was developed as IVD real-time PCR test kit for the analysis of *SHOX2* gene methylation in bisulfite converted DNA isolated from human bronchial lavage fluid. In 2017, the *Epi proLung^®^* blood-based lung cancer test received the CE-IVD mark, which is based on a combination of the methylation analyses of *SHOX2* and the prostaglandin E receptor 4 gene (*PTGER4*) [115,117].

Clearly, the ctDNA methylation patterns represent a powerful approach for early detection testing. The non-invasive ctDNA methylation biomarker assays could improve the compliance and early screening rates of head and neck cancer, where no such test has been developed so far.

A new diagnostic biomarker for early detection of HNC requires passing of all necessary phases of clinical trials before being approved. Despite the lack of standardized guidelines for clinical validity, risk-benefit, and clinical applications in regards to cancer screening, five crucial clinical phases should be considered with ctDNA-based liquid biopsy tests: (1) Pre-clinical exploratory phase aiming to identify promising directions, (2) Development and validation of clinical assays, which can detect an established disease, (3) Retrospective longitudinal phases to determine how well biomarkers detect preclinical disease by testing the markers in tissues collected prospectively from research cohorts, (4) Prospective screening in which the extent and characteristics of disease detected by the test are determined, as well as the false referral rate, and (5) Cancer control phase, which includes large-scale population studies to assess both the role of biomarkers in disease detection and the overall impact of screening in the population [118].

Ultimately, salivary detection *RASSF1A* hypermethylation appears to be a clear choice regarding the potential targets through liquid biopsy for further study and for implementation into clinical practice. Notably, the meta-analysis by Meng et al., which included 550 HNSCC tissues and 404 controls from 12 published studies, suggested a significant association (OR:2.93) between aberrant *RASSF1A* methylation in HNSCC [76]. Large-scale prospective studies are needed to confirm that salivary detection of *RASSF1A* hypermethylation could be a promising biomarker for an early HNSCC detection liquid biopsy-based test.

## 6. Conclusions

The presented review summarized the recent progress in early cancer detection, namely head and neck cancer (HNC), based on cfDNA. Standardized diagnostics are often not satisfactory in early HCN detection. On the other hand, cfDNA-based detection technology has already shown potential, albeit significant improvement is required to increase sensitivity to small amounts of cfDNA, especially in the case of early-stage cancer. Utilization of non-invasive liquid biopsy approaches significantly simplify the sample collection process, and the diagnostic results are easier to obtain and generally more reliable. Although it has been extensively studied and discussed in many published studies, validated clinical trials are urgently needed to demonstrate the extent of feasibility and effectiveness of the abovementioned early detection technologies in combination with standard-of-care screening modalities. Regarding effectiveness, safety, and minimal costs, future widespread distribution of this technology in preventive care may provide a significant advancement in early cancer detection.

## Figures and Tables

**Table 1:** Summary of DNA methylation markers in saliva of head and neck tumors.

Biomarker Name	Biological Function	Tumor Site	Sample Type	Sample Size	DNA Processing	Technical Approach (Methods)	Methylation Status	Application	Sensitivity (%)	Specificity (%)	AUC (95% CI)	Reference
*p16*	*Cell cycle regulation*	HNC (SCC)	Oral rinse (NaCl)	30 tumors and 30 saliva controls	Volume: - Kit: Phenol-chloroform extraction/Bisulfite treatment (Sigma, Burlington, MA, USA)	MSP	Hypermethylation	Diagnosis	NA	NA	NA	Rosas 2001 [34]
*MGMT*	*DNA repair*								NA	NA	NA	
*DAPK*	*Cell death regulation*								NA	NA	NA	
*TIMP3*	*Cell cycle regulation*	HNC (SCC)	Oral rinse (25 mL NaCl 0.9%)	60 patients	Volume: - Kit: QIAmp tissue kit (Qiagen, Hilden, Germany)/CpGenome DNA modification kit (MP Biomedicals, Irvine, CA, USA)	MSP	Hypermethylation	Diagnosis/ disease monitoring	28	NA	NA	Righini 2007 [35]
*ECAD*	*Cell adhesion*								20	NA	NA	
*p16*	*Cell cycle regulation*								27	NA	NA	
*MGMT*	*DNA repair*								22	NA	NA	
*DAPK*	*Cell death regulation*								15	NA	NA	
*RASSF1A*	*Cell cycle regulation*								17	NA	NA	
*soluble CD44*	*Cell adhesion*	HNC (SCC)	Oral rinse (5 mL NaCl, 5 s)	102 patients and 69 controls	Volume: - Kit: QIAmp DNA mini kit (Qiagen, Hilden, Germany)/bisulfite solution	MSP	Hypermethylation	Diagnosis	62–70	75–88	NA	Franzmann 2007 [36]
*p16*	*Cell cycle regulation*	OC (SCC)	Unstimulated saliva (7.5 mL)	14 patients and 5 controls	Volume: 1 mL Kit: QIAamp Blood (Qiagen, Hilden, Germany)/EpiTect Bisulfite (Qiagen, Hilden, Germany)	Methylight	Hypermethylation	Diagnosis	35	NA	NA	Viet 2007 [37]
*MGMT*	*DNA repair*								29	NA	NA	
*p15*	*Cell growth regulation, cell death regulation*								29	NA	NA	
*APC*	*Cell growth regulation*								14	NA	NA	
*ECAD*	*Cell adhesion*								7	NA	NA	
*GABRB3 + IL11 + INSR + NOTCH3 + NTRK3 + PXN*	*Cell signaling, cell differentiation, cell adhesion*	OC (SCC)	Unstimulated saliva (7.5 mL)	13 patients and 10 controls	Volume: 1 mL Kit:iPrep Chargeswitch Buccal Cell kit (Invitrogen, Waltham, MA, USA)/EZ DNA Methylation kit (Zymo Research, Irvine, CA, USA)	GoldenGate Methylation Array (Illumina, San Diego, CA, USA)	Hypermethylation	Diagnosis	77	87	NA	Viet 2008 [38]
*CCNA1*	*Cell cycle regulation*	HNC (SCC)	Oral rinse (20 mL NaCl)	175 patients and 444 controls	Volume: - Kit: Phenol-chloroform extraction/Bisulfite solution	qMSP	Hypermethylation	Diagnosis	20	97	>0.60	Carvalho 2008 [39]
*DAPK*	*Cell death regulation*			176 patients and 451 controls					16	96	>0.60	
*DCC*	*Cell cycle regulation*			176 patients and 462 controls					12	99	>0.60	
*MGMT*	*DNA repair*			149 patients and 239 controls					13	95	>0.60	
*TIMP3*	*Cell cycle regulation*			176 patients and 450 controls					11	93	>0.60	
*MINT31*	*Calcium channel regulator*			175 patients and 492 controls					5	100	>0.60	
*p16*	*Cell cycle regulation*			177 patients and 500 controls					5	100	>0.60	
*PGP9.5*	*Cell cycle regulation*			34 patients and 112 controls					82	30	>0.60	
*AIM1*	*Cell signaling*			23 patients and 73 controls					4	99	>0.60	
*ESR*	*Cell cycle regulation, cell signaling*			33 patients and 119 controls					3	98	>0.60	
*CCND2*	*Cell cycle regulation*			136 patients and 97 controls					7	90	>0.60	
*MINT1*	*Cell adhesion*			131 patients and 296 controls					35	66	>0.60	
*CDH1*	*Cell adhesion*			66 patients and 116 controls					30	38	>0.60	
*EDNRB*	*Cell signaling*	OC (SCC)	Oral rinse (25 mL NaCl, 15 s)	161 patients	Volume: - Kit: Phenol-chloroform extraction/EpiTect Bisulfite kit (Qiagen, Hilden, Germany)	qMSP	Hypermethylation	Diagnosis	65	51	0.61	Pattani 2010 [40]
*KIF1A*	*Cell signaling, extracellular transport*	HNC (SCC)	Oral rinse (20 mL NaCl)	71 patients and 61 controls	Volume: - Kit: Phenol-chloroform extraction/EpiTect Bisulfite kit (Qiagen, Hilden, Germany)	qMSP	Hypermethylation	Diagnosis	37	98	NA	Demokan 2010 [41]
*EDNRB*	*Cell signaling*								68	93	NA	
*KIF1A + EDNRB*	*-*								77	93	NA	
*HOXA9*	*Homeodomain control, cell differentiation*	OC (SCC)	Oral rinse (20 mL NaCl)	16 OC patients, 16 OPC patients and 19 controls	Volume: - Kit: Phenol-chloroform extraction/EpiTect Bisulfite kit (Qiagen, Hilden, Germany)	Discovery: Illumina Infinium HumanMethylation27 BeadChips. Validation: qMSP	Hypermethylation	Diagnosis	63	53	0.65	Guerrero-Preston 2011 [42]
*NID2*	*Cell adhesion, cell differentiation*								72	21	0.57	
*ECAD*	*Cell adhesion*	OC (SCC)	Oral rinse (20 mL NaCl, 30–60 s)	34 patients and 24 controls	Volume: 5 mL Kit: DNeasy Blood and Tissue (Qiagen, Hilden, Germany)/EpiTect Bisulfite (Qiagen, Hilden, Germany)	MSP	Hypermethylation	Diagnosis	94	80	0.91	Nagata 2012 [43]
*TMEFF2*	*Cell cycle regulation, cell differentiation, cell signaling*								85	87	0.90	
*RARß*	*Cell signaling, cell cycle regulation, cell differentiation*								82	92	0.88	
*MGMT*	*DNA repair*								77	80	0.81	
*FHIT*	*Cell death regulation*								80	67	0.75	
*WIF-1*	*Cell cycle regulation*								71	79	0.69	
*DAPK*	*Cell death regulation*								56	75	0.66	
*p16*	*Cell cycle regulation*								38	92	0.66	
*HIN-1*	*Cell cycle regulation, cell death regulation, cell growth regulation*								29	92	0.61	
*TIMP3*	*Cell cycle regulation*								24	96	0.60	
*p15*	*Cell growth regulation, cell death regulation*								65	63	0.58	
*APC*	*Cell growth regulation*								63	63	0.56	
*SPARC*	*Cell adhesion, cell differentiation*								41	67	0.51	
*ECAD + TMEFF2 + RARB + MGMT*	*-*								100	88	NA	
*ECAD + TMEFF2 + MGMT*	*-*								97	92	NA	
*ECAD + TMEFF2 + RARB*	*-*								94	96	NA	
*ECAD + RARB + MGMT*	*-*								91	92	NA	
*DAPK*	*Cell death regulation*	OC (SCC)	Oral rinse (4 mL NaCl)	77 oral precancer patients and 32 OC(SCC) samples	Volume: - Kit: Phenol-chloroform extraction/Bisulfite solution	qMSP	Hypermethylation	Diagnosis	3	NA	NA	Liu 2012 [44]
*p16*	*Cell cycle regulation*	OC (SCC)	Oral rinse (16 mL NaCl, 30 s)	10 patients and 3 controls	Volume: 3 mL Kit: Methylamp Whole Cell Bisulfite Modification (Epigentek, Farmingdale, NY, USA)	MSP	Hypermethylation	Diagnosis	40	100	NA	Kusumoto 2012 [45]
*p16^INK4a^*	*Cell cycle regulation*	HNC (SCC)	Unstimulated saliva (DNA-SAL Salivary DNA Collection Device)	143 patients and 46 controls	Volume: - Kit: EpiTect Plus Kit (Qiagen, Hilden, Germany)	Nested MSP	Hypermethylation	Diagnosis	58	91	NA	Ovchinnikov 2012 [46]
*RASSF1A*	*Cell cycle regulation*								55	80	NA	
*DAPK1*	*Cell death regulation*								13	98	NA	
*p16^INK4a^ + RASSF1A + DAPK1*	*-*								80	87	NA	
*DCC*	*Cell cycle regulation*	HNC (SCC)	Oral rinse (10 mL NaCl 0.9%)	146 pretreated patients and 60 controls	Volume: - Kit: Phenol-chloroform extraction/Bisulfite solution	qMSP	Hypermethylation	Diagnosis	52	90	NA	Rettori 2013 [47]
*CCNA1*	*Cell cycle regulation*								11	97	NA	
*DAPK*	*Cell death regulation*								8	98	NA	
*MGMT*	*DNA repair*								8	97	NA	
*TIMP3*	*Cell cycle regulation*								5	98	NA	
*MINT31*	*Calcium channel regulator*								4	100	NA	
*AIM1*	*Cell signaling*								3	100	NA	
*SFRP1*	*Cell growth regulation, cell differentiation*								3	100	NA	
*APC*	*Cell growth regulation*								3	100	NA	
*p16*	*Cell cycle regulation*								1	100	NA	
*HIN-1*	*Cell cycle regulation, cell death regulation, cell growth regulation*								12	81	NA	
*CCNA1 + DAPK + DCC + MGMT + TIMP3*	*-*								55	76	NA	
*CCNA1 + DAPK + MGMT + TIMP3*	*-*								20	82	NA	
*CCNA1 + DAPK + MGMT*	*-*								18	85	NA	
*CCNA1 + MGMT + TIMP3*	*-*								18	85	NA	
*CCNA1 + DAPK + TIMP3*	*-*								16	92	NA	
*DAPK + MGMT + TIMP3*	*-*								16	85	NA	
*CCNA1 + MGMT*	*-*								16	88	NA	
*CCNA1 + DAPK*	*-*								15	95	NA	
*CCNA1 + TIMP3*	*-*								14	93	NA	
*Alu*	*Cell cycle regulation, cell signaling*	OC (SCC)	Oral rinse (10 mL NaCl 0.9%, 15 s)	43 patients and 108 controls	Volume: - Kit: Phenol-chloroform extraction/Bisulfite solution	COBRA	Hypomethylation	Diagnosis	87	57	NA	Puttipanyalears 2013 [48]
*EDNRB*	*Cell signaling*	HNC (SCC)	Oral rinse (20 mL NaCl)	191 patients	Volume: - Kit: Phenol-chloroform extraction/EpiTect Bisulfite kit (Qiagen, Hilden, Germany)	qMSP	Hypermethylation	Diagnosis	73	51	0.65	Schussel 2013 [49]
*DCC*	*Cell cycle regulation*								69	59	0.65	
*EDNRB + DCC*	*-*								75	48	0.67	
*MED15/PCQAP3′*	*Cell cycle regulation*	HNC (SCC)	Unstimulated saliva	44 patients and 45 controls	Volume: - Kit: EpiTect Plus Kit (Qiagen, Hilden, Germany)	MSP	Hypermethylation	Diagnosis	68	58	0.63	Ovchinnikov 2014 [50]
*MED15/PCQAP5′*				46 patients and 49 controls					70	63	0.70	
*ZNF14*	*Cell cycle regulation*	HNC (SCC)	Oral rinse	59 patients and 35 controls	Volume: 250 μL Kit: Phenol-chloroform extraction/EpiTect Bisulfite kit (Qiagen, Hilden, Germany)	Discovery: Illumina Infinium HumanMethylation27 BeadChips. Validation: qMSP	Hypermethylation	Diagnosis	8	100	NA	Gaykalova 2015 [51]
*ZNF160*	*Cell cycle regulation*								17	100	NA	
*ZNF420*	*Cell cycle regulation, cell death regulation*								14	100	NA	
*RASSF1α + p16^INK4a^ + TIMP3 + PCQAP5′* + *PCQAP3′*	*-*	HNC (SCC)	Unstimulated saliva	88 HPV- patients and 122 controls	Volume: - Kit: The Epitect Plus DNA Bisulfite Kit (Qiagen, Hilden, Germany)	MSP	Hypermethylation	Diagnosis	71	80	0.86	Lim 2016 [52]
				45 HPV+ patients and 122 controls					80	74	0.80	
*p16*	*Cell cycle regulation*	OC (SCC)	Saliva (Oragene^®^ DNA Self-Collection kit)	58 patients and 90 controls	Volume: - Kit: Oragene^®^ DNA/Bisulfite treatment (Sigma, Burlington, MA, USA)	MSP	Hypermethylation	Diagnosis	17	94	NA	Ferlazzo 2017 [53]
*MGMT*	*DNA repair*								28	92	NA	
*p16 + MGMT*	*-*								21	NA	NA	
*ZNF582*	*Cell cycle regulation*	OC (SCC)	Oral rinse (20 mL mouth rinse solution containing 0.12% chlorhexidine, 20 s)	94 patients and 65 controls	Volume: 0.4 mL Kit: Epigene Nucleic Acid Extraction (iStat Biomedical, Taipei City, Taiwan)/Bisulfite conversion (iStat Biomedical, Taipei City, Taiwan)	qMSP	Hypermethylation	Diagnosis	65	75	NA	Cheng 2018 [54]
*PAX1*	*Cell differentiation*								64	82	NA	
*TRH*	*Cell cycle regulation, thyroid hormone regulation*	OC (SCC)	Oral rinse (10 mL 0.9% NaCl, 15 s)	42 patients and 54 controls	Volume: - Kit: QIAamp DNA FFPE Tissue (Qiagen, Hilden, Germany)/EZ DNA Methylation-Gold (Zymo Research, Irvine, CA, USA)	qMSP	Hypermethylation	Diagnosis	88	93	0.93	Puttipanyalears 2018 [55]
		OPC (SCC)		24 patients and 54 controls					83	93	0.88	
*p16 + RASSF1α + TIMP3 + PCQAP/MED15*	*-*	OC (SCC)	Unstimulated saliva (2 mL)	54 OC patients, 34 OPC patients and 60 controls	Volume: - Kit: DNeasy Blood and Tissue (Qiagen, Hilden, Germany)/EpiTect Plus DNA Bisulfite (Qiagen, Hilden, Germany)	MSP	Hypermethylation	Diagnosis	92	92	0.92	Liyanage 2019 [56]
		OPC (SCC)							100	92	0.97	
*NID2*	*Cell adhesion, cell differentiation*	OC (SCC)	Oral rinse (0.9% NaCl, 15 s)	43 patients and 90 controls	Volume: - Kit: Phenol-chloroform extraction/EZ DNA Methylation (Zymo Research, Irvine, CA, USA)	qMSP	Hypermethylation	Diagnosis	79	100	NA	Srisuttee 2020 [57]
*EDNRB*	*Cell signaling*	OPC (SCC)	Oral rinse (15 mL NaCl)	21 patients and 40 controls	Volume: - Kit: EpiTect Plus DNA Bisulfite (Qiagen, Hilden, Germany)	qMSP	Hypermethylation	Diagnosis/recurence detection	72	95	0.83	Shen 2020 [58]
*PAX5*	*Cell differentiation, cell cycle regulation*								70	91	0.78	
*p16^INK4a^*	*Cell cycle regulation*								17	100	0.59	
*p16*	*Cell cycle regulation*	OC (SCC)	Unstimulated saliva (5 mL)	43 patients and 40 controls	Volume: 200 μL Kit: QIAamp DNA blood mini kit (Qiagen, Hilden, Germany)/Epitect Plus DNA Bisulfite Kit (Qiagen, Hilden, Germany)	MSP	Hypermethylation	Diagnosis	44	90	NA	Rapado-González 2021 [59]
*RASSF1A*	*Cell cycle regulation*								23	95	NA	
*p16 + RASSF1A*	*-*								54	88	NA	

*Abbreviations*: HNC = head and neck cancer; HNSCC = head and neck squamous cell carcinoma; OC = oral cancer; OSCC = oral squamous cell carcinoma; OPC = oropharyngeal cancer; HPV = human papilloma virus; MSP = methylation-specific polymerase chain reaction; qMSP = quantitative MSP.
